# Posterior fixation of gastric tube with fibrin sealant in laparoscopic sleeve gastrectomy: a promising method to prevent revision surgeries

**DOI:** 10.1007/s00423-024-03253-8

**Published:** 2024-02-14

**Authors:** Mehmet Celal Kizilkaya, Ridvan Gokay, Arda Ulaş Mutlu, Suleyman Sonmez, Serhan Yilmaz, Ali Kocatas, Can Saracoglu, Erman Aytac

**Affiliations:** 1 Acıbadem University Atakent Hospital, Department of General Surgery, Istanbul, Turkey; 2 Luleburgaz State Hospital, Kırklareli, Turkey; 3 University of Health Sciences Istanbul Kanuni Sultan Süleyman Training and Research Hospital, Istanbul, Turkey

**Keywords:** Laparoscopic sleeve gastrectomy, Posterior fixation, Fibrin glue, Revisional surgery, Weight regain

## Abstract

**Background:**

We aim to assess the effects of gastric posterior fixation with fibrin sealant in laparoscopic sleeve gastrectomy in aspects of 12th-month body mass index and gastric volume.

**Methods:**

The patients who underwent laparoscopic sleeve gastrectomy between January 2019 and February 2021 were divided into two groups preoperatively. The first 75 patients were appointed to the posterior fixation group, and the second 75 were to the control group. Changes in gastric volume and body mass index were assessed in the postoperative 12th month.

**Results:**

There were 110 patients in the final analysis. Fifty-four patients had posterior fixation, and 56 had only laparoscopic sleeve gastrectomy. The posterior fixation group was superior in terms of total weight loss rate (39.1% vs. 34.5%, *p*<0.001) and less gastric volume increase rate (39.8% vs. 164.7%, *p*<0.001) in the postoperative 12th month.

**Conclusion:**

Our study suggests that posterior fixation with fibrin sealant in laparoscopic sleeve gastrectomy is a promising method for preventing weight regain and creating a need for revision surgery.

## Introduction

The laparoscopic sleeve gastrectomy (LSG) is the most performed surgical treatment of obesity due to its safety and efficacy [1]. The primary rationale behind this surgery is minimizing the gastric volume (GV) and causing a physical restriction with maximum safety and effectiveness [2]. Even though it is considered an efficient method, more than a quarter of the patients regain weight [3].

The persistence of obesity is the reason for 87% of bariatric revision surgeries and 12.2% of obese patients undergoing revision surgery in the following 10 years after the LSG [4]. Since revision surgery increases the risks of short-term complications, different techniques are tried to decrease revision surgery necessity. An FDA-approved material, fibrin sealant, is one of the materials that is used in bariatric surgery to prevent short-term and long-term complications. It has been used in many studies in the literature that fibrin sealant prevents leakage, bleeding, vomiting, dysphagia, and adhesion-related twisting [5].

It is known that fibrin glue was used as a supporting material in many cases in the literature. [5] Posterior fixation with fibrin glue preserves the gastric curve more efficiently in the postoperative term. This preservation ensures a better postoperative recovery with less morbidity and more weight loss. [5, 6] Thus, posterior fixation is a favorable factor for the improvement of quality of life and success of the surgery.

It is hypothesized that the increase in remaining gastric tissue’s volume would be less since posterior fixation adheres the stomach from the staple line to the neighboring posterior tissues. This study aims to evaluate the effects of posterior fixation with fibrin sealant in LSG on body mass index (BMI) and GV.

## Materials and methods

Data from the patient who underwent LSG in our hospital between January 2019 and February 2021 were collected prospectively. Patients were divided into LSG + posterior fixation (PF) and LSG only. The first 75 patients presented to our clinic were included in the LSG+PF group, and the following 75 patients were included in the LSG-only group. Forty patients were excluded from the study due to loss of follow-up. Fifty-four patients in LSG+PF and 56 in LSG only group were included in the final analysis.

**Fig. 1:**
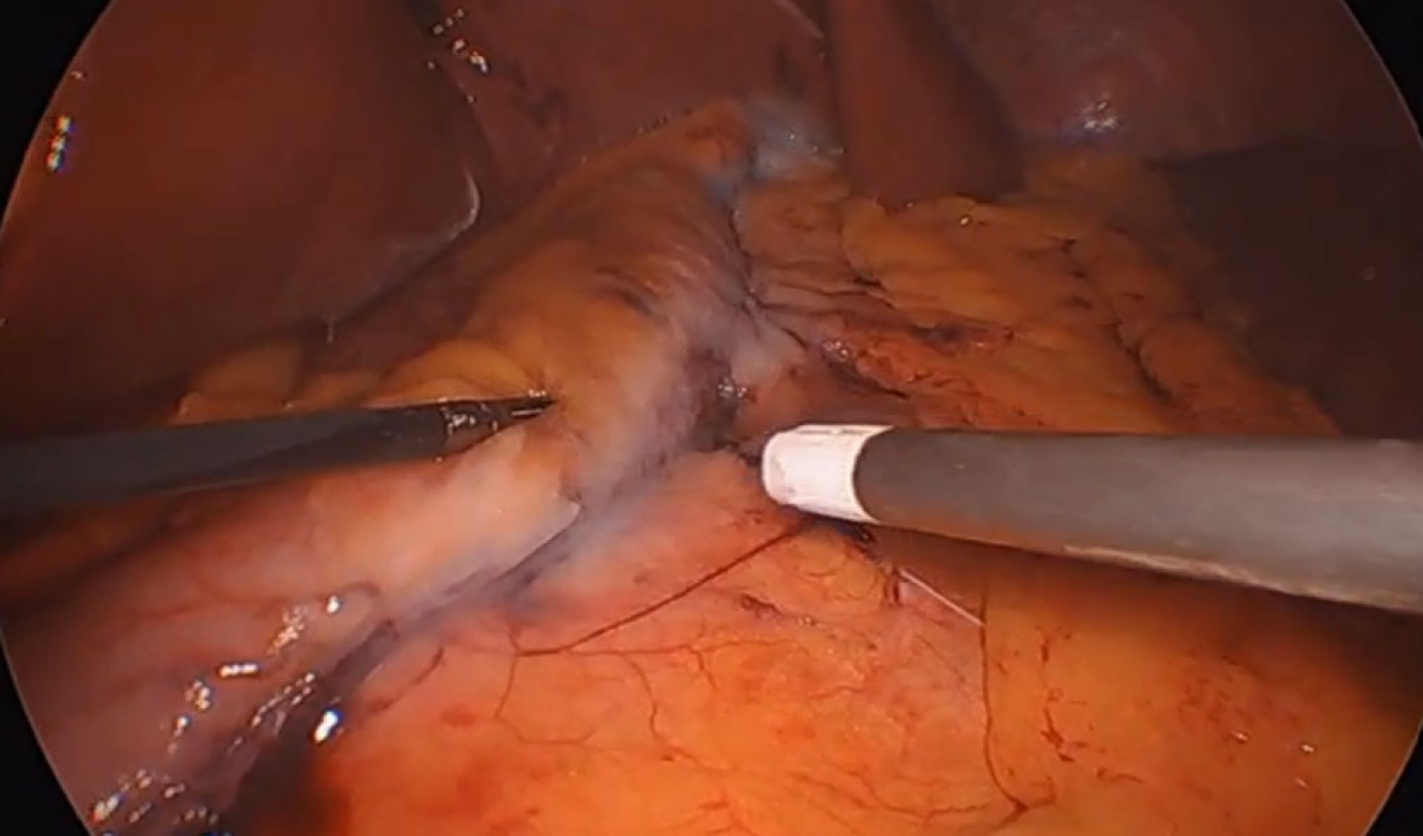
Intraoperative image following the injection of fibrin sealant

The same surgeon operated on all of the patients. Following the transection, bleeding control, and leakage test with methylene blue, the stomach was placed anatomically. In the LSG+PF group, 4 ml of fibrin glue (Tisseel, Baxter, Deerfield, Illinois, USA) were applied to the posterior surface of the remaining gastric tissue starting from the stapler line (Fig. 1).

**Fig. 2:**
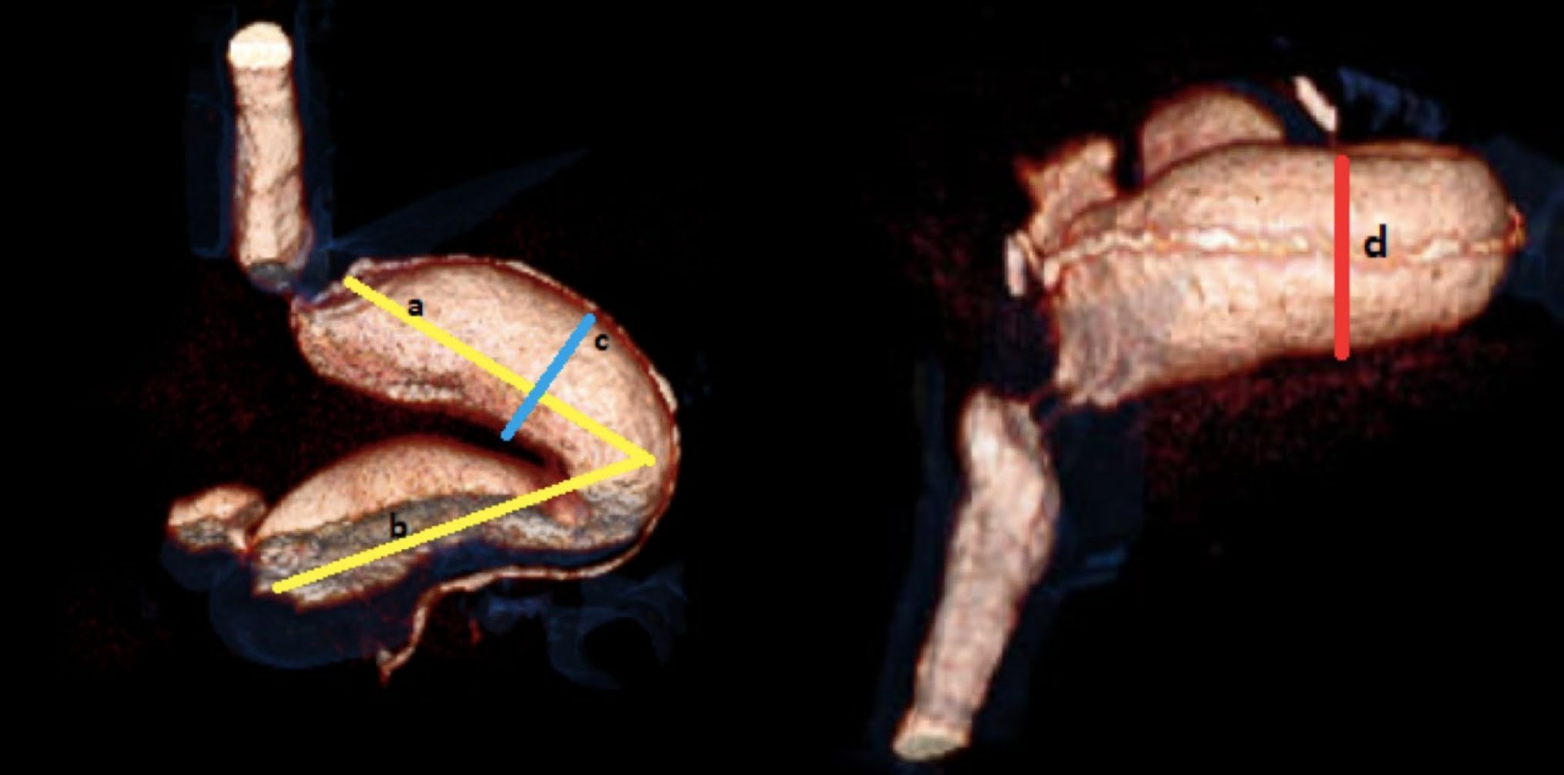
Dimensional computed tomography image of gastric volume calculation

For the analysis of the GV change, computed tomography imaging in the postoperative 2nd day and 12th-month were used. The same experienced gastrointestinal diagnostic radiologist did radiological assessments. The remaining gastric tissue’s volume was calculated using length, width, and depth calculated from 2D and 3D images. Following the calculations, all three diameters were multiplied (length*width*depth)cm^3^, and the result was considered as the remaining gastric volume (Fig. 2).

The percent change in GV was calculated with $\left[\frac{\textrm{Initial}\ \textrm{GV}-12\textrm{th}\ \textrm{Month}\ \textrm{GV}}{\textrm{Initial}\ \textrm{GV}}\times 100\right]$, and the decrease in BMI was calculated with $\hbox{--} \left[\frac{\textrm{Initial}\ \textrm{BMI}-12\textrm{th}\ \textrm{Month}\ \textrm{BMI}}{\textrm{Initial}\ \textrm{BMI}}\times 100\right]$. Results greater than zero were accepted as an increase in GV and a decrease in BMI.

The chi-square (*x*^2^) test was used to compare the categorical variables between the two groups. Mann–Whitney *U* and Student *t* tests were used to compare the numerical variables. Paired samples test and Wilcoxon signed rank test were used to compare the continuous variables. IBM SPSS Statistics for Windows, Version 22.0, was used for the statistical analyses.

## Results

One hundred ten patients were included in the final analysis. 56 (50.9%) patients underwent LSG+FP, while the remaining patients underwent LSG only. 101 (91.8%) patients were female. The median age of the patients was 36 years. 105 (95.5%) patients had class III obesity, and the remaining had class II obesity on their first visits. 35 (31.8%) patients had at least 1 obesity-related comorbidity preoperatively. In total, 19 (17.3%) patients had 1 comorbidity, 13 (11.8%) patients had 2, 2 (1.8%) patients had 3, and 1 (0.9%) patient had 4 obesity-related comorbidities. No difference was seen in gender, age, initial obesity class, or obesity-related morbidity between the two groups (*p*=0.74, 0.471, 0.676, and 0.223, respectively). Patient characteristics are shown in Table 1.

**Table 1:** Patient characteristics

	Total (*n*=110)	LSG+PF (*n*=56)	LSG only (*n*=54)	*p*
Gender (female)	101 (91.8%)	52 (92.9%)	49 (90.7%)	0.740
Age, median (IQR)	35 (15)	36 (19)	34 (12)	0.471
Initial obesity (class III)	105 (95.5%)	54 (96.4%)	51 (94.4%)	0.676
Initial comorbidity (0)	75 (68.2%)	35 (62.5%)	40 (74.1%)	0.223

Initial BMI and postoperative 2nd-day GV were similar in both groups, *p*=0.48 and *p*=0.082, respectively. LSG+PF group patients’ postoperative 12th-month BMI was lower, and absolute BMI reduction was significantly higher compared to the LSG-only group (27.82 kg/m^2^ vs. 29.4 kg/m^2^, *p*=0.031 and 17.9 vs. 15.05, *p*=0.001, respectively). The total weight loss rate was higher in the LSG+PF group (39.13% vs. 34.5%, *p*<0.001, respectively). In the postoperative 12-month follow-up, the LSG-only group was prone to have a more GV increase rate (164.72% vs.39.76%, *p*=0.001, respectively). The details on BMI and GV are presented in Table 2.

**Table 2:** Weight and gastric volume

	Total (*n*=110)	LSG+PF (*n*=56)	LSG only (*n*=54)	*p*
Initial GV, median (IQR)	48.04 (19.65)	48.77 (16.14)	43.99 (23.36)	0.082
1st-year GV, median (IQR)	91.18 (63.39)	70.64 (45.74)	114.66 (53.32)	**<0.001**
GV increase rate, median (IQR)	112.84 (145.3)	39.76 (92.76)	164.72 (125.64)	**<0.001**
Initial BMI (kg/m^2^), median (IQR)	44.85 (6.58)	45.6 (8.08)	44.05 (6.65)	0.480
1st-year BMI (kg/m^2^), median (IQR)	28.6±3.95	27.83±3.91	29.41±3.86	0.036*
%TWL	36.86±7.39	39.13±6.69	34.5±7.4	<0.001*

*GV* Gastric volume, *BMI* body mass index, *TWL* total weight loss, *Student *t* test

## Discussion

This study reports the 12-month follow-up of the patients who underwent LSG with and without PF. Obesity is a chronic disease that causes various comorbidities [7]. In our study, 35 (31.8%) patients had at least one obesity-related comorbidity. Even lifestyle change is a crucial necessity for the treatment of obesity and obesity-related comorbidities, different surgical approaches can be used to treat obese patients when the lifestyle change is not enough alone [8].

LSG is the most preferred surgical approach for treating obesity and obesity-related comorbidities [1]. The surgery causes biochemical and mechanical changes, resulting in increased satiety and decreased calorie intake. The 7-year follow-up results of SLEEVEPASS Trial [9] showed that patients with LSG lose 47% (95% CI, 43–50%) of their initial weight. The percent total weight loss (%TWL) in our patients’ postoperative 12th-month follow-up was 36.85% (95% CI, 35.46–38.26%).

Abdallah et al. reported the differences between 2 and 6 cm from the pylorus antrectomy [10]. In the postoperative 12th-month follow-up, the percent excess weight loss (%EWL) was significantly higher in the 2-cm group (63.8% vs. 51.9%, *p*=0.0001). Following the 2-cm approach, the mean %TWL in our series was 36.8. In the same study by Abdallah et al. [10], gastric leakage and late vomiting rates of the 2-cm group were 3.8 and 13.5, respectively. In our study, no patient had postoperative gastric leakage or late vomiting.

Weight loss is a complex process that includes medical, psychological, and lifestyle factors. Although this complexity brings different parameters that affect the result, it is reported that the initial surgical technique is the most contributing factor in obesity treatment [11]. Ferrer-Marquez et al. [12] reported a significant increase in gastric volume from postoperative 1st to 12th month (68.39 vs. 122.58 cm^3^, *p*<0.001). This increase in gastric volume had no significant correlation with the percent excess body mass index loss (*r* = 0.01; *p*= 0.910). In complete contrast, our study showed a significant increase in weight loss parallel to less gastric volume increase in LSG+PF group.

The sleeve gastrectomy is a promising method in obesity treatment. Still, the long-term follow-up studies show that many of the patients require another revision surgery due to different causes [13]. A study showed that the ratio of revision surgery requirement increases up to 15.3% in the postoperative 8th year [14]. A national study by Lazzati et al. [4] reported that 87% of revision bariatric surgeries are caused by weight regain. Our study showed that the LSG+PF group had a significantly higher reduction in BMI and a lower rate of GV increase compared to LSG only group. Considering that weight regain is the primary cause of revision surgeries, our PF technique can decrease the revision surgery rates by reducing the weight regain [4].

Fibrin sealant is a United States Food and Drug Administration approved hemostat, cement, and adhesive material since 1998 [15]. Its adhesive effects in tissue grafts were reported in a study by Uğur et al. [16] in patients with distal vaginal agenesis. The usage of FS in bariatric surgery was first described in the management of patients with gastric leakage following gastric bypass surgery [17]. FS was efficient in treating 7 patients with leakage. Although these patients required more than one FS injection, it proved the material’s efficacy on gastric tissue adhesion. Coşkun et al. [5] reported a 1000-case series with FS in LSG. They reported better hemostasis for the stapler line and fewer complications, including leakage, twist, and stricture. Kızılkaya et al. [6] described that FS injection has resulted in fewer complications (2.1% vs. 11.7%, *p*=0.008) and a lower BMI (28 kg/m^2^ vs. 32.8 kg/m^2^, *p*<0.001) in the postoperative 6th-month. Similar to the aforementioned study, the LSG+PF group had a better weight reduction compared to the LSG-only group. In addition, the LSG+PF group had a significantly smaller GV in the postoperative 12th month.

Predicting the outcome of the surgery by the RGV is a widely used technique. Different methods can be used to calculate the RGV. Pilone et al. evaluated their patients with 3D CT scans and lower RGV was significantly associated with a higher %EWL in the postoperative 12th month (31.9% vs. 51.8%, *p*<0.05) [18]. Doğan et al. [19] used intraoperative CO_2_ inflation to measure the RGV and it did not show any significant relationship with weight loss in the postoperative 6th, 12th, and 24th months. In our study, we used the 3D CT scan to measure the RGV, and it was statistically significant with weight loss.

The rationale behind the LSG is GV reduction and limiting the nutritional intake. Thus, the effects of postoperative GV are widely researched in LSG. The initial sleeve size is reported not to affect weight loss in the short-term but is associated with increased weight regain in long-term follow-up [20]. Hanssen et al. [21] reported the correlation between postoperative 6th-month GV and %EWL. These two parameters were inversely correlated (*r*^2^=0.316, *p*=0.001). Thus, postoperative GV can be used safely in indicating surgical success and lost weight protection. The LSG+PF group had a nonsignificant bigger GV in the postoperative 2nd day and higher BMI postoperatively. Even though these results are poor prognostic factors, the LSG+PF group had superior 12th-month results.

## Conclusion

Posterior fixation in LSG is promising for higher weight loss and lower GV increase rate. Its role in surgery is a safer and more efficient treatment. Our study reports the first 12th-month of follow-up in patients with PF. The major limitation of this study is the lack of reporting the long-term result of this newly described technique, and long-term results will be reported in the future using the data of the patients who completed the follow-up.

## References

[1] Angrisani L, Santonicola A, Iovino P, Vitiello A, Higa K, Himpens J (2018). IFSO Worldwide Survey 2016: primary, endoluminal, and revisional procedures. Obes Surg..

[2] Vidal P, Ramón JM, Goday A, Benaiges D, Trillo L, Parri A (2013). Laparoscopic gastric bypass versus laparoscopic sleeve gastrectomy as a definitive surgical procedure for morbid obesity. Mid-term results. Obes Surg.

[3] Casella G, Soricelli E, Giannotti D, Collalti M, Maselli R, Genco A (2016). Long-term results after laparoscopic sleeve gastrectomy in a large monocentric series. Surg Obes Relat Dis.

[4] Lazzati A, Bechet S, Jouma S, Paolino L, Jung C (2020). Revision surgery after sleeve gastrectomy: a nationwide study with 10 years of follow-up. Surg Obes Relat Dis.

[5] Coskun H, Yardimci E (2017). Effects and results of fibrin sealant use in 1000 laparoscopic sleeve gastrectomy cases. Surg Endosc.

[6] Kizilkaya MC, Bozkurt MA (2022). New technique of posterior fixation of tube with fibrin sealant prevents dysphagia in patients undergoing sleeve gastrectomy. Am Surg.

[7] Jung UJ, Choi MS (2014). Obesity and its metabolic complications: the role of adipokines and the relationship between obesity, inflammation, insulin resistance, dyslipidemia and nonalcoholic fatty liver disease. Int J Mol Sci.

[8] Clifton PM (2012). Bariatric surgery: effects on the metabolic complications of obesity. Curr Atheroscler Rep.

[9] Grönroos S, Helmiö M, Juuti A, Tiusanen R, Hurme S, Löyttyniemi E (2021). Effect of laparoscopic sleeve gastrectomy vs Roux-en-Y gastric bypass on weight loss and quality of life at 7 years in patients with morbid obesity: the SLEEVEPASS randomized clinical trial. JAMA Surg.

[10] Abdallah E, El Nakeeb A, Yousef T, Abdallah H, Ellatif MA, Lotfy A (2014). Impact of extent of antral resection on surgical outcomes of sleeve gastrectomy for morbid obesity (a prospective randomized study). Obes Surg.

[11] Lauti M, Kularatna M, Hill AG, MacCormick AD (2016). Weight regain following sleeve gastrectomy—a systematic review. Obes Surg.

[12] Ferrer-Márquez M, García-Díaz JJ, Moreno-Serrano A, García-Díez JM, Ferrer-Ayza M, Alarcón-Rodríguez R (2017). Changes in gastric volume and their implications for weight loss after laparoscopic sleeve gastrectomy. Obes Surg.

[13] English WJ, EJ DM, Brethauer SA, Mattar SG, Rosenthal RJ, Morton JM (2018). American Society for Metabolic and Bariatric Surgery estimation of metabolic and bariatric procedures performed in the United States in 2016. Surg Obes Relat Dis.

[14] Tsui ST, Yang J, Nie L, Altieri MS, Talamini M, Pryor AD (2020). Association of revisions or conversions after sleeve gastrectomy with annual bariatric center procedural volume in the state of New York. Surg Endosc.

[15] Spotnitz WD (2014). Fibrin sealant: the only approved hemostat, sealant, and adhesive—a laboratory and clinical perspective. ISRN Surg.

[16] Ugur MG, Balat O, Ozturk E, Bekerecioglu M, Dikensoy E (2012). Pitfalls in diagnosis and management of distal vaginal agenesis: 10-year experience at a single centre. Eur J Obstet Gynecol Reprod Biol.

[17] Brolin RE, Lin JM (2013). Treatment of gastric leaks after Roux-en-Y gastric bypass: a paradigm shift. Surg Obes Relat Dis.

[18] Pilone V, Tramontano S, Cutolo C, Griguolo G, Di Spirito F, Pagano AM (2020). Relation of gastric volume with weight loss after sleeve gastrectomy: results of a prospective analysis. Surg Laparosc Endosc Percutan Tech.

[19] Doğan S, Önmez A, Çetin MF, Özaydın İ, Pehlivan M (2020). Residual gastric volume relationship and weight loss after laparoscopic sleeve gastrectomy. Obes Surg.

[20] Weiner RA, Weiner S, Pomhoff I, Jacobi C, Makarewicz W, Weigand G (2007). Laparoscopic sleeve gastrectomy--influence of sleeve size and resected gastric volume. Obes Surg.

[21] Hanssen A, Plotnikov S, Acosta G, Nuñez JT, Haddad J, Rodriguez C (2018). 3D volumetry and its correlation between postoperative gastric volume and excess weight loss after sleeve gastrectomy. Obes Surg.

